# Error in Figure 2

**DOI:** 10.1001/jamanetworkopen.2021.44317

**Published:** 2021-12-22

In the Original Investigation titled “Pivotal Evaluation of an Artificial Intelligence System for Autonomous Detection of Referrable and Vision-Threatening Diabetic Retinopathy,”^1^ published November 15, 2021, there was a wording error in Figure 2. The wording in the flow diagram given as “intent-to-treat” should have been “intent-to-screen.” This article has been corrected.^1^
